# The UK Centre for Astrobiology: A Virtual Astrobiology Centre. Accomplishments and Lessons Learned, 2011–2016

**DOI:** 10.1089/ast.2017.1713

**Published:** 2018-02-01

**Authors:** Charles S. Cockell, Beth Biller, Casey Bryce, Claire Cousins, Susana Direito, Duncan Forgan, Mark Fox-Powell, Jesse Harrison, Hanna Landenmark, Sophie Nixon, Samuel J. Payler, Ken Rice, Toby Samuels, Petra Schwendner, Adam Stevens, Natasha Nicholson, Jennifer Wadsworth

**Affiliations:** ^1^ UK Centre for Astrobiology, Scottish Universities Physics Alliance, School of Physics and Astronomy, The University of Edinburgh, Edinburgh, UK.; ^2^ Eberhard Karls Universitaet Tuebingen, Center for Applied Geoscience (ZAG), Geomicrobiology, Tuebingen, Germany.; ^3^ Centre for Exoplanet Science, SUPA, School of Physics and Astronomy, University of St Andrews, St Andrews, UK.; ^4^ Division of Microbial Ecology, Department of Microbiology and Ecosystem Science, Research Network “Chemistry Meets Microbiology”, University of Vienna, Vienna, Austria.; ^5^ Geomicrobiology Research Group, School of Earth, Atmospheric and Environmental Sciences, University of Manchester, Manchester, UK.; ^6^ Institute of Evolutionary Biology, School of Biological Sciences, University of Edinburgh, Edinburgh, UK.

## Abstract

The UK Centre for Astrobiology (UKCA) was set up in 2011 as a virtual center to contribute to astrobiology research, education, and outreach. After 5 years, we describe this center and its work in each of these areas. Its research has focused on studying life in extreme environments, the limits of life on Earth, and implications for habitability elsewhere. Among its research infrastructure projects, UKCA has assembled an underground astrobiology laboratory that has hosted a deep subsurface planetary analog program, and it has developed new flow-through systems to study extraterrestrial aqueous environments. UKCA has used this research backdrop to develop education programs in astrobiology, including a massive open online course in astrobiology that has attracted over 120,000 students, a teacher training program, and an initiative to take astrobiology into prisons. In this paper, we review these activities and others with a particular focus on providing lessons to others who may consider setting up an astrobiology center, institute, or science facility. We discuss experience in integrating astrobiology research into teaching and education activities. Key Words: Astrobiology—Centre—Education—Subsurface—Analog research. Astrobiology 18, 224–243.

## 1. Introduction

Establishing a center in any type of research is a nontrivial task in organization. Astrobiology brings with it a particular set of challenges. As a field, it is highly interdisciplinary, cutting across physics/astrophysics, chemistry, biology, geosciences, and other fields (Des Marais *et al.,* 2003; Smith *et al.,* 2013). Some of its scientific questions involve philosophy and social sciences (Chela-Flores, 2011; Race *et al.,* 2012), particularly those that deal with the human presence in space and the launching of craft to planetary bodies that may host habitable conditions. A major challenge in the development of astrobiology is the successful integration of these fields.

Despite these challenges, astrobiology is a hugely rewarding area of science. Its interdisciplinary remit brings traditionally separated subject areas together, often forming some of the richest areas of science. In addition to primary scientific research, it offers enormous potential in its contribution to education (Sauterer, 2000; Quinlan, 2015). One of its most potent questions—*“Are we alone in the Universe?”*—fascinates people of all ages and interests. Thus, astrobiology is a vehicle for teaching fundamental science with questions that inspire interest of benefit to the traditional school curriculum and the general public.

In 2011, we set up a virtual astrobiology center, the UK Centre for Astrobiology (UKCA), situated at the University of Edinburgh (www.astrobiology.ac.uk). Over the past 5 years, we have garnered a substantial cross section of experience in how to develop such a center and in the various ways of developing astrobiology in research, technology, and educational activities. After 5 years, we share our experiences, encourage others to set up similar centers, and point out experiences and approaches that we think may be useful.

## 2. The UK Centre for Astrobiology: Initial Rationale and Objectives

**FIG. 1.**
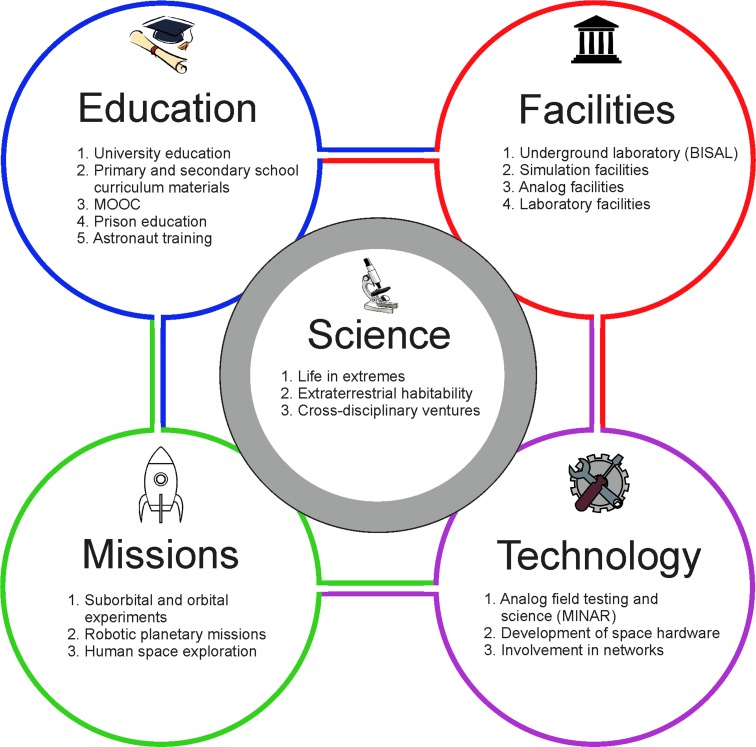
Structure of the UK Centre for Astrobiology showing the links between its initiatives.

We established UKCA within the School of Physics and Astronomy at the University of Edinburgh in late 2011. Its overall aim was to make a contribution to advancing astrobiology in the United Kingdom and internationally. The specific objectives of UKCA were defined as a desire to contribute to astrobiology in three main areas: (1) science, (2) technology development, and (3) education and outreach. We established a set of projects in each of these three areas that have been our focus for the first 5 years (Fig. 1).

The strategic objectives of UKCA were summarized in its mission statement, which was defined as follows:

The mission of the UK Centre for Astrobiology is to advance knowledge of molecules and life in extreme environments on the Earth and beyond to further our understanding of planetary habitability. It does this with a combination of theoretical, laboratory, field and mission approaches. We apply this knowledge to improving the quality of life on Earth and developing space exploration as two mutually enhancing objectives.

From the beginning, UKCA was primarily envisaged as a virtual organization, focusing on the study of life in extreme environments and its applications to investigating extraterrestrial habitability. It became an international partner of the NASA Astrobiology Institute (NAI) in 2012. Although UKCA began its activities in late 2011, it was officially opened in April 2013 in an event at the National Museum of Scotland.

The UK Centre for Astrobiology has been run by a local Board at the University of Edinburgh that oversees its day-to-day activities. It has also assembled a National Board to advise on wider integration of UKCA at the UK level.

## 3. Research

**FIG. 2.**
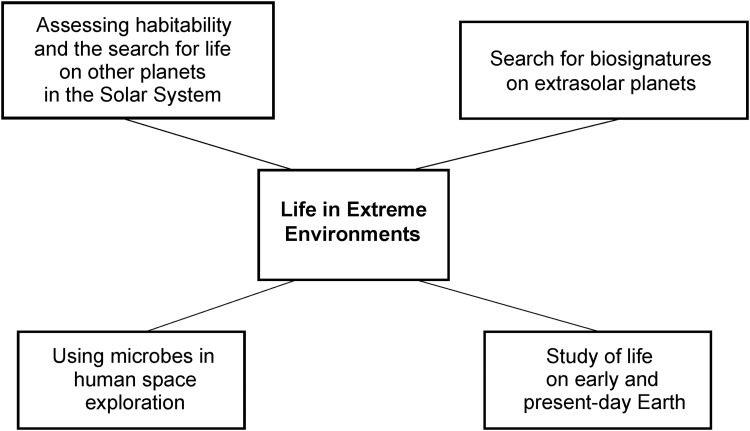
The study of life in extreme environments is relevant to many areas of astrobiology.

From the start of UKCA's activities, it was recognized that the scope of possibilities in astrobiology is enormous and no single group can hope to cover all aspects of astrobiology in equal and significant depth. The expertise that we had within the group that established UKCA was the study of microbial life in extreme environments (Fig. 2). Microbiology is a subject that cuts across many facets of astrobiology. If we are to successfully assay other planets for life, improve our ability to search for life in the rock record, understand how life has persisted in the planetary crust for over 3.5 billion years, or if we are to seek biosignatures in the atmospheres of exoplanets, it is to terrestrial biology that we must turn. More specifically, microbiology forms the core of this activity since microbes dominate the diversity and biomass of life in many environments and, in particular, extreme environments. Thus, scientific questions concerning the microbiology of extreme environments are a core area of scientific investigation in understanding biology in its cosmic context more generally.

We therefore arranged our research activity at UKCA around some key science questions that fit into the general two themes of life in extremes and the habitability of extraterrestrial environments. This organizational rationale has been the driver for determining the projects that UKCA has engaged in and is reflected in the mission statement.

The work has been accomplished by a core of 8 postdoctoral scientists and 16 PhD students over a 5-year period and with a large number of national and international collaborations linked into the effort as well as collaborations within Edinburgh. In addition, Fellows have been funded directly through UKCA (for example by the Royal Society of Edinburgh, the EU Marie-Curie initiative, and the US National Science Foundation), and visiting honorary fellow positions have been used to carry out collaborative projects in this area. The greatest challenge is ensuring continuous coordination of these efforts. We have accomplished this by regular meetings, on average once a week.

### 3.1. Life in extremes

We have found it effective to arrange our research under a set of specific science questions, and here we provide examples of this research.

**FIG. 3.**
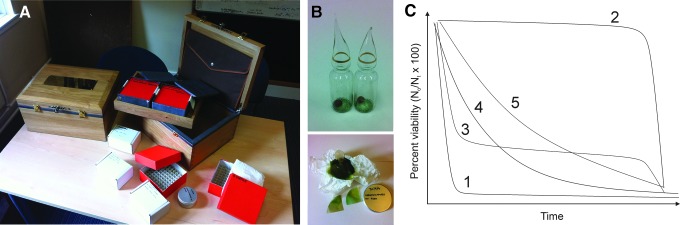
The 500-year microbiology experiment. (**A**) The experiment investigates the loss of viability and biochemical changes in *Bacillus subtilis* and *Chroococcidiopsis* over century time periods. (**B**) Microorganisms are desiccated in vials. Sheets of *Chroococcidiopsis* desiccated on agar are also included. (**C**) One purpose of the experiment is to investigate the function that describes the decline of organisms over time.

A major question is *What are the limits of microbial growth, and how do microbes adapt to extremes?* Work has included the following:

(1) A combination of laboratory-based and theoretical approaches (Harrison *et al.,* 2013; Freeman *et al.,* 2016). A particular focus for this work has been to understand how combinations of multiple extremes shape the “boundary space” for microbial cell division, whether on a planetary scale or within a selected ecosystem (or ecosystems) (Harrison *et al.,* 2015a, 2015b), which advances beyond our understanding of single extreme limits (Cockell and Nixon, 2013).
(2) Studies on adaptations of life to low temperatures (Perfumo *et al.,* 2014; Nixon *et al.,* 2017) and radiation (Wadsworth and Cockell, 2017).
(3) Collaborations on investigating the lower water activity limit of life (Stevenson *et al.,* 2015).
(4) Investigations on interactions of microbes and antibiotics in extreme conditions (Harrison *et al.,* 2017).
(5) Studies on the consequences of microbial and organismal biomass for the total biomass on Earth (Landenmark *et al.,* 2015).
(6) Implementation of a long-term science experiment (Fig. 3)—the 500-year microbiology experiment—to study how microbial viability is lost over multicentury timescales (Cockell, 2014b; Cockell *et al.,* 2015c),

A logical development of the first question is *What adaptations have allowed microbes to persist in the planetary crust for over 3.5 billion years?* Work we have undertaken that falls within this purview has included:

(i) studies on the molecular (proteomic) responses of microbes to extreme conditions living within the planetary crust (Bryce *et al.,* 2016);
(ii) studies on the colonization of extreme volcanic environments (Kelly *et al.,* 2014; Cockell *et al.,* 2015b);
(iii) studies on habitats in impact craters (Pontefract *et al.,* 2014, 2016); and
(iv) UKCA involvement, through science party membership, in the IODP (International Ocean Discovery Program) Expedition 364 to drill the peak ring of the Chicxulub impact crater (Morgan *et al.,* 2016), linked to the end-Cretaceous mass extinction (Schulte *et al.,* 2010). UKCA work in this project has been focused on understanding how impact craters produce habitats for deep subsurface life. The work has implications for the habitability of the deep subsurface of other planetary bodies influenced by the effects of impacts.

### 3.2. Extraterrestrial habitability

The UK Centre for Astrobiology is interested in using its study of life in extremes on Earth to understand the potential habitability of extraterrestrial environments (Cockell, 2014a,d, 2015a). One science question that has focused our work is *Are extraterrestrial aqueous environments habitable?* For example, simulated martian brines have been used to probe the limits of life within them and infer the importance of parameters other than water activity, such as ionic strength, in determining habitability in martian brines (Fox-Powell *et al.,* 2016). Work has investigated the habitability of pH-neutral environments prevalent during the Noachian on Mars and the plausible redox couples for life, including the iron cycle (Nixon *et al.,* 2012). UKCA has sought to understand what factors might limit microbial metabolisms on Mars, such as the presence of microbial toxins (Nixon *et al.,* 2013; Nixon and Cockell, 2015). UKCA has been involved in using data from Mars missions, such as the NASA Mars Science Laboratory (Curiosity rover), to understand the habitability of present-day Mars (Martin-Torres *et al.,* 2015).

A second science question of interest relates to the behavior and application of microbes in space, such as on orbital platforms. It can be summarized as *How do microbes adapt to or survive in space conditions?* (Moissl-Eichinger *et al.,* 2016). Members of UKCA have been involved in numerous space experiments, such as in the ESA EXPOSE-E experiment in which the outside environment of the International Space Station was used to investigate impact-shocked rocks as habitats to protect microorganisms from high UV radiation on anoxic planets (Bryce *et al.,* 2015). More recently, in the EXPOSE-R2 (BOSS and BIOMEX) International Space Station experiments, UKCA investigated the survival of an extreme tolerant cyanobacterium (*Gloeocapsa* sp.) in space conditions that we previously isolated from rocks flown in space (Olsson-Francis *et al.,* 2010; Cockell *et al.,* 2011). The experiment BOSS (PI: Petra Rettberg, DLR) has allowed us to study the way in which the biofilm habit of the organism contributes to its resistance to extreme conditions. The experiment BIOMEX (PI: Jean-Paul de Vera, DLR) has allowed us to study the role of iron in both causing damage and providing protection to microorganisms.

**FIG. 4.**
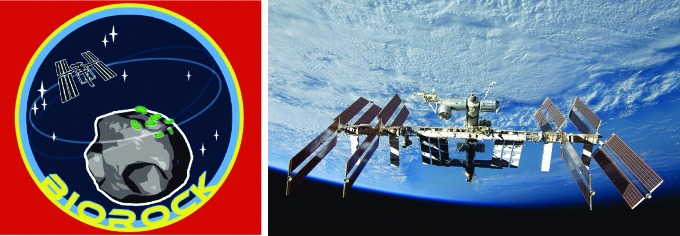
BioRock is an ESA-approved experiment due to fly on the International Space Station and part of a wider interest in space microbiology experiments.

The UK Centre for Astrobiology is science coordinator of the project BioRock (Fig. 4), an ESA-approved experiment for the International Space Station. The project involves the study of biofilms and microbe-mineral interactions in microgravity and simulated martian gravity using the Kubik centrifuge. The project is focused on a better understanding of how microbes behave and form biofilms in space, with their potential applications to improving life support, extraction of minerals from rocks, and other uses of microbes in space exploration (Raafat *et al.*, 2013; Loudon *et al.,* 2018). In addition to orbital studies, the experiment has also involved testing on parabolic flights with ESA.

The UK Centre for Astrobiology was the lead organization on the ESA Geobiology in Space Exploration (GESE) Topical team (Cousins *et al.,* 2015), a group established with funding from ESA for 3 years to consider the applied use of microbes in space with a special focus on the use of microbe-mineral interactions. This 3-year UKCA-led phase was a continuation of a prior 3-year iteration of this network (Cockell, 2010).

The third question that derives from the research articulated in the previous two questions is *How can this knowledge be used to assist in the search for life elsewhere?* With this question, we have sought to link our research into planetary missions. Members of UKCA have been involved in the development of instrumentation and science related to instruments proposed or planned for space missions, particularly where these innovations link to investigations on life in extreme environments. Some of these projects have included involvement in the science or science teams of the Close-Up Imager (CLUPI) (Josset *et al.,* 2017), the HABIT instrument (HabitAbility: Brine, Irradiation and Temperature) selected for the ExoMars mission, the development of a robotic hammer (SPLIT), and the SOLID (Signs of Life Detector) instrument being developed for a range of future space missions. Members of UKCA have been involved in the ESA Mars Trace Gas Orbiter mission. We have also used our study of extremophile microbes to investigate their preservation potential, for example, studies on the effects of salinity on the fossilization of cells (Harrison *et al.,* 2016).

Beyond our own solar system, UKCA's research on life in extreme environments can be used to inform predictions about the habitability of exoplanets (Fig. 2), the potential life they might sustain, and how we might detect it. UKCA has taken special interest in exoplanet research that directly links to microbiology and the use of known limits of life to inform ideas about the habitability of exoplanets (O'Malley-James *et al.,* 2013, 2014, 2015; Brown *et al.,* 2014; Cockell, 2014c; Forgan *et al.,* 2015; Schwieterman *et al.,* 2015, 2018; Yates *et al.,* 2017), and it has applied its expertise to considering galactic habitability (Forgan *et al.,* 2017).

### 3.3. Cross-disciplinary international efforts

One advantage we have found of having a clear strategic direction and a set of well-defined science questions is that they can be used to fashion projects or lead projects that bring the science questions together into integrated efforts. The critical mass established to address the science questions has made it easier to contribute to projects where cross-disciplinary links are important.

**FIG. 5.**
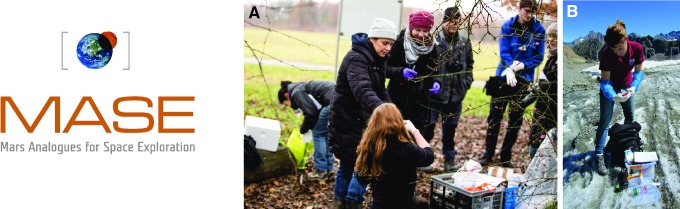
MASE is an EU project focused on the study of anaerobic microorganisms from Mars analog environments. Anoxic collection of microorganisms in (**A**) sulfide springs in Germany and **(B**) glaciers in Austria.

For example, within Europe, UKCA has led scientific coordination in a project funded through the European Union (EU), entitled MASE (Mars Analogues for Space Exploration) (Fig. 5), whose partners were the European Science Foundation (ESF), DLR (Germany), Universidad Autonoma de Madrid (Spain), the Spanish Centre for Astrobiology (Spain), the University of Leiden (Netherlands), CNRS (France), the University of Regensburg (Germany), the University of Graz (Austria), Matis (Iceland), and the European Astrobiology Network Association (EANA, Europe-wide).

Anaerobic organisms have not been investigated as much as aerobic organisms, yet most extraterrestrial environments are anoxic. The objective of MASE was originally to isolate novel anaerobic microorganisms from a diversity of Mars analog sites and achieve their deposition within a culture collection (DSMZ) so that they can be used by other scientists. Subsequently, the project has used these organisms to undertake fundamental studies on their responses to Mars-relevant extremes and their potential for preservation during fossilization (*e.g.,* Beblo-Vranesevic *et al.,* 2017; Cockell *et al.,* 2017; Gaboyer *et al.,* 2017). The project has also set about to test spacecraft instruments on these preserved organisms to better determine the limits of the detection of life. All of these segments of the project fall under the previous science questions that were identified. MASE is an example of an international project that cuts across all of our defined science questions in a coordination role at the European level.

A second example of international collaboration linked to our affiliate status of the NAI is our work within the NASA-funded project BASALT (Biologic Analog Science Associated with Lava Terrains).

**FIG. 6.**
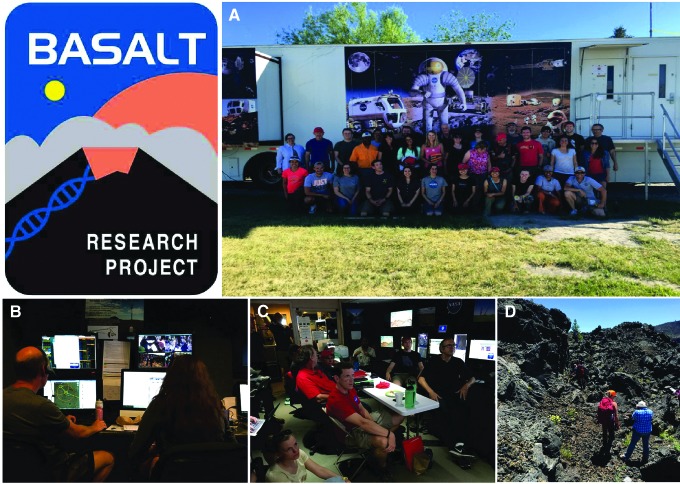
NASA's BASALT analog program seeks to develop protocols for the human exploration of Mars and understand life in volcanic environments. (**A**) The team deployed in the Craters of the Moon in Idaho, June 2016. (**B**) Remote operations with the EVA team in the field. (**C**) During the project, mission protocols are developed for human planetary exploration. (**D**) The field work involves the geological, geochemical, and biological study of basaltic rocks.

BASALT is a large analog program run from NASA Ames Research Center (PI: Darlene Lim) under NASA's Planetary Science and Technology Analog Research Program (PSTAR) (Fig. 6). Its aim is to develop exploration, logistics, and communications protocols for the human exploration of Mars by running full-scale EVA (extravehicular activity) simulations in the lava fields of Idaho and Hawaii over a series of campaigns. Embedded within this effort is the collection of basaltic rocks from diverse weathering and alteration types to study microbial communities. Volcanic terrains form the majority of the martian surface (Cousins *et al.,* 2013; Cousins, 2015; Harris *et al.,* 2015); thus the investigation of their microbiology on Earth is key to understanding their habitability elsewhere in addition to advancing our understanding of the microbiology and biogeochemistry of these terrains on Earth.

The UK Centre for Astrobiology's role within BASALT was as lead organization in the study of the microbial communities. Our objective was to understand how microbial communities persist and what sorts of metabolisms exist in lava terrains to improve our understanding of the limits of life in volcanic environments on Earth and our ability to assess the habitability of Mars. This objective links directly into UKCA's science and technology focus.

## 4. Astrobiology Community Building

One role of a virtual organization can be to help build the community of researchers by bringing them together in different permutations designed to encourage interaction, thus helping to encourage new collaboration and the strengthening of the community. Motivated by an interest in using the critical mass of researchers at Edinburgh, we have hosted a diversity of meetings. Two initiatives were focused on building new community structures, as follows.

(1) In 2014, UKCA hosted a 1-day meeting entitled Building Habitable Worlds, aimed at bringing together PhD students, postdocs, and other early-career scientists across the United Kingdom, Europe, and beyond. The motivation for doing this was to generate a highly informal meeting during which early career scientists (who may not otherwise meet easily) could get to know each other's work across very diverse disciplines, discuss collaboration, and stay in touch during the course of their studies.

The first half-day was given to 2–3 min pitches from all the participants to briefly explain their research interests. The second half of the day was focused on grouping the participants together around tables and having them come up with research ideas and collaborations that would bring their various interests together with a view to continuing collaboration after the event. This format was successful. As an example, one group conceived of an idea in the afternoon discussion, continued collaborating immediately after the event, and subsequently published a paper (Stevens *et al.,* 2016). This paper examined the potential of detecting atmospheric signatures of past civilizations. This meeting was repeated at the University of Glasgow in February 2015 and returned to Edinburgh in February 2017. Each of these meetings attracted between 30 and 45 participants.

(2) The second example was the inaugural meeting of AbGradE, European Astrobiology Graduates in Europe (Noack *et al.,* 2015; Samuels *et al.,* 2015) in Edinburgh in 2014. The meeting involved presented talks and discussion and attracted 41 participants from six nations across Europe as well as the United States and Mexico. The motivation behind AbGradE was to bring together early-career researchers across Europe and foster closer collaboration between new astrobiologists. Since the inaugural meeting, it has been held prior to each EANA meeting in Europe.

In addition to new meetings, a center can also act as a convenient node for bringing the astrobiology community together in existing formats. Examples of the meetings we have held are (a) *meetings to contribute to international- and national-level astrobiology* (*e.g.,* a 3-day “workshop-without walls” on “The Present Habitability of Mars” at UCLA, co-hosted with the University of California Los Angeles [UCLA in 2013], the European astrobiology conference [EANA] in 2014, and the 5^th^ Astrobiology Society of Britain [ASB] conference in April 2013); (b) *meetings for space agencies* (*e.g.,* ESA's Planetary Protection Working Group, November 2012); (c) *meetings to develop specific scientific networks* (*e.g.,* workshops of the STFC-funded network GeoRepNet, focused on identifying science and technology challenges in establishing geological repositories, and a meeting of the ESA Geobiology in Space Exploration [GESE] Topical team [Cousins *et al.,* 2015] in Sardinia in May 2015).

The UK Centre for Astrobiology has also run an internal University of Edinburgh seminar series in astrobiology and regular coffee meetings (“habitability coffee”) for researchers at Edinburgh interested in astrobiology. We have found these initiatives helpful in bringing astrobiology researchers together at the university, strengthening the profile of UKCA within Edinburgh, and providing a forum for early-career researchers to meet.

Although organizing meetings can be labor-intensive, we found that organizing them provided early-career researchers with an opportunity to demonstrate a range of useful skills on their CVs including managing meetings, coordinating international logistics, and planning research discussions, all of which help equip them to demonstrate their capacity to lead research activity. Thus, delegated in the right way (for example, to PhD students in the middle of their studies as opposed to those starting their PhD work or writing up, so as to minimize disruption), these meetings have enormous benefit not just to the participants but also to the organizers. We also recommend that early-career researchers be allowed to take leadership of meetings. Our first Building Habitable Worlds conference was conceived by two PhD students and a postdoctoral scientist, and they were given free rein to organize it in their own style, while UKCA provided logistical and financial help. The result was a meeting with which early-career researchers were strongly engaged.

## 5. Research Infrastructure

One useful contribution a center can make is to establish infrastructure that can be used by other research groups. UKCA focused its efforts on developing an underground astrobiology laboratory and extraterrestrial aqueous environment simulation capabilities. The laboratory project was motivated by the potentially exciting science questions and technology testing that could be accomplished by using an existing underground science facility at the Boulby Mine, UK. We viewed it as a valuable way to add an analog field capability to our chosen science questions and create a research facility that UKCA could offer to a wide range of research groups. The building of simulation facilities was motivated by a desire to create new facilities that would allow for extraterrestrial environments to be simulated. Both were consistent with our science focus on life in extreme environments and habitability.

### 5.1. Underground astrobiology laboratory

The deep subsurface is recognized to host a high abundance of microorganisms (Kallmeyer and Wagner, 2014), and it may be a location for habitable conditions on Mars (Boston *et al.,* 1992). Understanding how life grows and persists in deep, dark, often energy-depleted environments is therefore important for understanding biogeochemistry on Earth and the search for habitable conditions elsewhere. These environments were of interest in the context of UKCA's defined science questions.

The Science and Technology Facilities Council (STFC) in the United Kingdom has been running an underground science facility at Boulby Mine since 2005 (De Angelis, 2017). Boulby Mine is an active potash/polyhalite mine in the northeast of the United Kingdom that operates over 1000 km of roadways at a depth of ∼1 km underground. The mine operates in vast evaporite deposits that are ∼0.25 billion years old and were formed in the Zechstein Sea, an inland epicontinental sea that existed in the Permian Period (298.9–252.17 million years ago) (*e.g.,* Taylor, 1998; Zhang *et al.,* 2013).

**FIG. 7.**
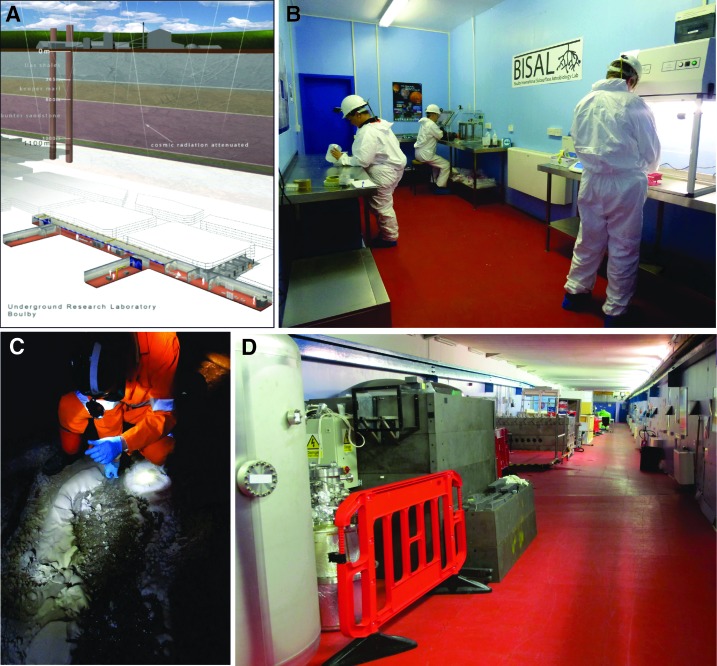
An underground astrobiology laboratory. (**A**) The STFC Underground Science Facility at 1.1 km depth at Boulby Mine. (**B**) The mine hosts BISAL. (**C**) The laboratory can be used, among many other activities, to study life in the deep subsurface. (**D**) It also offers the potential for longer-term studies of life at below-background radiation in conjunction with the other low-background radiation facilities developed in the laboratory.

The underground science facility was originally focused on dark matter detection but has expanded its remit to other areas of physics and geosciences. In 2011, UKCA approached the facility with a concept for the world's first permanent underground astrobiology laboratory focused on studying life in the deep subsurface, an initiative that resulted in BISAL (Boulby International Subsurface Astrobiology Laboratory) (Fig. 7) (Cockell *et al.,* 2013). The laboratory is a microbiology lab equipped for basic microbiology procedures under aseptic conditions. It provides a base from which to carry out studies of deep subsurface biology and geochemistry as well as a facility for clean analog activities (see Section 6.1).

To date, studies in BISAL have included a range of activities that link back to our core science questions: (1) isolation and characterization of novel anaerobic microbes under the aegis of the EU MASE project, (2) environmental genomic and metagenomic studies of brines of differing composition, (3) investigation of the viable microbiome and biosignatures in Permian salts, (4) analysis of gases associated with Permian salts, (5) the study of microbial motility in the deep subsurface, and (6) the study of life at below-background radiation. Some of these studies do not require the fixed laboratory but are undertaken with simple sample collection trips. Nevertheless, the laboratory provides the focus for the whole range of subsurface astrobiology activities, some of which require a long-term presence.

Our experiences at Boulby illustrate a general point that significant astrobiology capabilities can be developed in parallel with existing facilities or by leveraging existing resources. To assemble an underground astrobiology laboratory from scratch would cost a very large amount of money. However, in aligning BISAL with the existing STFC-funded underground facility, we achieved the establishment of a piece of infrastructure at low cost that has ongoing support in the milieu of the other projects at the site.

### 5.2. Extraterrestrial aqueous environment simulation

Some of the most important advances in astrobiology over the last few decades have been the confirmation of aqueous extraterrestrial environments. As liquid water is thought to be a requirement for life (at least as we know it), the search for active aqueous extraterrestrial environments takes on special significance. Although evidence for sustained bodies of liquid water on Mars was first suspected in Viking images, the discovery of phyllosilicates, high-resolution images of past lakes and rivers, and direct imaging of sedimentary deposits on Mars confirm that Mars hosted long-lived water bodies and potentially habitable conditions (Grotzinger *et al.,* 2014). Orbital evidence has confirmed the presence of subsurface oceans on Europa and Enceladus (Khurana *et al.,* 1998; Hand and Chyba, 2007; Postberg *et al.,* 2009; Waite *et al.,* 2009). Other bodies are likely to have subsurface oceans, including Ganymede and Titan (Lorenz *et al.,* 2008; Vance *et al.,* 2014).

**FIG. 8.**
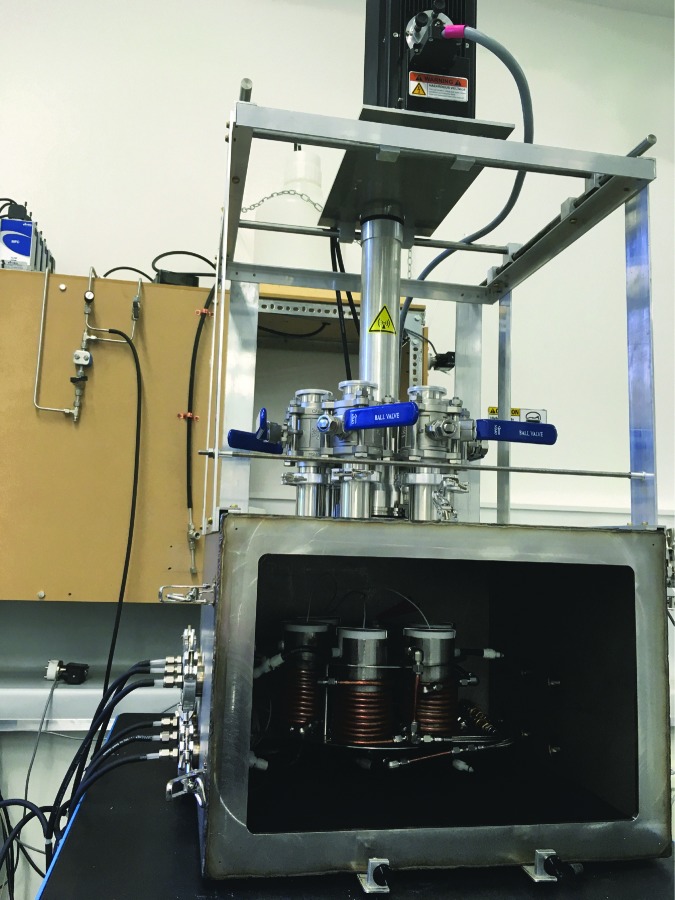
The development of new extraterrestrial simulation facilities, such as the PELS (Planetary Environment Liquid Simulator) can be used to carry out new studies on the habitability of extraterrestrial environments.

All of these discoveries show that investigating the habitability of extraterrestrial aqueous environments has become critical to advancing our understanding of life's potential beyond Earth. One enormous challenge in the laboratory is simulating these anaerobic environments. In 2011, we began construction of a Mars flow-through simulator, the first such chamber to allow for long-term aqueous experiments under Mars-simulated conditions in which liquid and solid sample acquisition could be accomplished without breaking atmospheric conditions (Martin and Cockell, 2015). Since then we have begun efforts to construct deep subsurface (high pressure) simulation facilities (Fig. 8). The motivation behind this work is to generate facilities that can be used by other researchers who use UKCA as a node. These facilities align with our science questions in understanding the habitability of extraterrestrial aqueous environments and the space environment in general.

## 6. Technology Development and Transfer

In the process of carrying out research activities, a center can also contribute to technology development. A motivation for doing this is to enhance societal impact and, specifically in the case of astrobiology, to help develop technology relevant to space missions.

### 6.1. Mine Analogue Research (MINAR)

Having built an underground laboratory, it was apparent that we could leverage our activity to contribute to technology transfer from astrobiology to other areas of social benefit.

Many of the challenges faced by planetary scientists (such as building small miniaturized instruments that are lightweight, require low energy, and operate in extremes) are also challenges faced by those in the deep subsurface mining sector who want instruments to detect and quantify ore quality, map and study subsurface tunnels and structures, and check underground environments for safety. A deep subsurface mine therefore provides an opportunity to carry out deep subsurface planetary science work in a real commercial setting where ideas can fluidly move from scientists and instrument developers to the mining environment.

From the perspective of planetary exploration, the deep subsurface is the most likely place to host habitable conditions on Mars (Fernández-Remolar *et al.,* 2008), and the discovery of lava tubes on the Moon (Coombs and Hawke, 1992) and Mars (Cushing *et al.,* 2007) suggests these places might be potential locations to access the subsurface of these bodies for geological studies. Eventually, they may be preferred locations for human-tended stations. Thus, deep subsurface environments on Earth, including mines, offer the potential to advance our understanding of the geology and habitability of extraterrestrial deep subsurface environments.

**FIG. 9.**
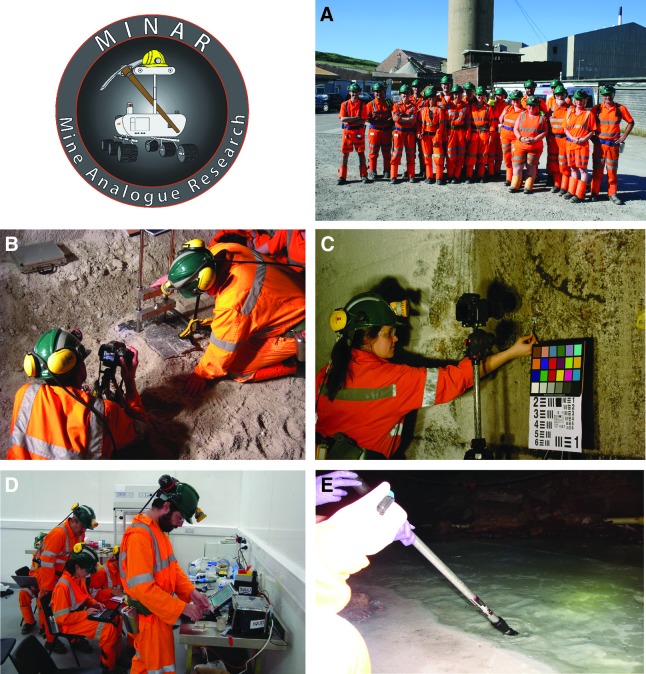
The MINAR program uses the Boulby Mine in the United Kingdom as a deep subsurface analog site. (**A**) The MINAR 4 team (2016) from the European Science Foundation, the Spanish Centre for Astrobiology, the University of Leicester, the University of Edinburgh, and Universidad Autonoma de Madrid. (**B**) Testing a robotic geologist's hammer (SPLIT) (University of Leicester). (**C**) Testing of the Panoramic Emulator Camera (PanCam) (University of Aberystwyth). (**D**) Testing of SOLID (Signs of Life Detector) in the underground astrobiology laboratory (BISAL) (Spanish Centre for Astrobiology). (**E**) Collecting anoxic sediments to characterize the microbial communities in the framework of the EU project MASE.

In 2013, we established a program called MINAR (Mine Analogue Research), which uses the scientific backdrop of our research activity and our existing underground work at the Boulby Mine as a deep subsurface analog site to test instrumentation for planetary exploration and/or deep subsurface work, study the deep biosphere, and act as a focus for other analog activities (Fig. 9). The analog work is a means by which to introduce planetary scientists to an active mining environment and encourage cross-fertilization of ideas between planetary scientists and the mining environment.

MINAR began with a workshop (From Outer Space to Mining) in April 2013 to scope possible analog activities (Bowler, 2013). The first three MINAR campaigns ran from 2013 to 2015 and involved coordinated testing of a wide diversity of instruments being developed for planetary exploration. They included instrumentation associated with the European ExoMars lander: CLUPI (Close-Up Imager), Raman, and PanCam emulator. Many other instruments were tested including proposed spacecraft XRD/XRF instruments, next-generation ultrasonic drills, miniature microscopy methods, a robotic geologist's hammer (SPLIT), and others. The details of this cross-collaboration for MINAR 1–3 can be found in the work of Payler *et al.* (2016).

MINAR 4 occurred in 2016 and was focused on the study of biosignatures in ancient salts in a collaboration with NASA Ames Research Center, the Spanish Centre for Astrobiology, and the EU MASE project. It was focused on the study of biosignatures in the Permian salt and their detectability with both lab-based and spacecraft instrumentation. The campaign also investigated the use of off-the-shelf microscopes and endoscopes for exploration purposes in mines and other planetary environments. MINAR 1–4 have involved between 15 and 30 individuals.

MINAR 5 will occur in 2017 in collaboration with Spaceward Bound, an educational program organized at NASA Ames Research Center, and will involve a 2-week analog campaign involving research, technology testing, and education and outreach. Our education effort will focus on lesson plan development for primary and secondary schools and India-wide education through live links from the subsurface mine to schools in India, with the support of the Kalam Centre in New Delhi. ESA astronaut Matthias Maurer will take part in the initiative with a focus on developing sampling methods and education.

Analog sites provide an opportunity for field research and contribution to the global availability of analog sites in support of Earth sciences and planetary exploration. In our case, Boulby was the first site in the United Kingdom to be seriously developed as an extraterrestrial analog testing environment, thus providing an easily accessible site to UK researchers and at the same time providing an opportunity for instrument developers to test technology in a working mine environment. Where a center can get access to or create an analog environment, this can provide a field component to its activities and a locus for involving international teams in collaboration with the center. Scientists from nine European countries and the United States have used the MINAR program to test instrumentation and carry out research.

### 6.2. Geological Repositories Network

Members of UKCA were involved in the establishment and management of an international network designated GeoRepNet (Geological Repositories Network), a 3-year network (2013–2016), funded by the STFC Futures Programme. Its purpose was to investigate the major scientific and technical challenges in the establishment and monitoring of geological repositories, which is a generic term for underground facilities used to store waste, such as nuclear waste or carbon capture and storage. This network was established to identify scientific priorities relevant to geological repositories (in the areas of geology, geochemistry, geophysics, and geobiology) and the technical implications (including instrument development and monitoring). In a series of meetings, key challenges in understanding the processes that influence the establishment, operation, and monitoring of geological repositories were identified and prioritized. An outcome was the identification of technology transfer and instrument development from the space and physics sector into geological repositories (Direito *et al.,* 2015). This network also provided funding in the form of travel, early career, and instrumentation awards in order to advance science and technology collaborations related to deep subsurface processes and geological repositories.

The motivation for carrying out GeoRepNet was the possibility of contributing to technology transfer from deep subsurface science and technology (consistent with our science goals and the existing BISAL and MINAR activity in Boulby).

## 7. Social Sciences

One of astrobiology's enduring and interesting characteristics is that it cuts across social sciences. Many of its questions, such as the ethics of contaminating habitable environments on other planetary bodies, the social consequences of finding extraterrestrial life, or the future of humans in space, involve philosophical and ethical dimensions. We found that using a center to engage in the social science dimension considerably enriched our activities and brought researchers in the humanities into contact with astrobiology questions.

Related to the study of life in extreme environments, and consistent with UKCA's focus, is the future of humans in the extreme environments of space. Perhaps one of the most profound social questions that underpins the human presence in extreme environments beyond Earth is how much freedom people can experience under these extremes. On the one hand, space is a limitless infinite frontier that offers enormous possibilities for expansion. However, we can also ask: Is it possible for people to experience any form of freedom in this environment when they are restricted to enclosed habitats and spacesuits? Can you be free when someone else controls the manufacture of oxygen on which you depend for survival on a second-to-second timescale? The question of what constitutes freedom concerned the ancient Greeks (Thucydides, BCE 431); it was approached with great vigor during The Enlightenment (*e.g.,* Hobbes, 1651; Locke, 1689; Rousseau, 1762) and has sat at the core of almost every major struggle, civil and military, in human history.

The UK Centre for Astrobiology launched a set of three workshops in collaboration with the British Interplanetary Society on the subject of Extraterrestrial Liberty. The first of these workshops, in June 2013, considered what freedom meant beyond Earth, exploring the way in which the space environment might change our perception of what freedom is and how the extreme conditions in space might erode some forms of liberty. The second workshop, in June 2014, considered the policy implications of these findings. How would the influence of space on freedom change human governance on the space frontier, and what are the political and economic implications of attempting to incorporate ideas of freedom into human governance? The final workshop, in June 2015, considered how denizens of the space frontier could change governance structures they disagreed with. Is revolution possible in space without enormous damage? What are the best ways to diffuse dissent in an environment where damage to infrastructure could kill many people? How does one achieve rational evolutionary change in human institutions in extreme environments?

These workshops were designed to bring together (i) planetary scientists and astrobiologists who have knowledge of life in extremes and the physical extremities of the space frontier, (ii) philosophers and political philosophers who have knowledge of the formal arguments and approaches relevant to the problem of freedom, (iii) science fiction writers who have knowledge of the substantial corpus of fiction that sometimes addresses the nature of liberty beyond Earth, and (iv) other individuals with an interest in the subject who wished to attend or contribute to subsequent publications.

The three workshops led to a trilogy of books published by Springer under their Space and Society series. The books, *The Meaning of Liberty Beyond Earth, Human Governance Beyond Earth: Implications for Freedom,* and *Dissent, Revolution and Liberty Beyond Earth* (Cockell, 2015a, 2015b, 2016) contained a total of 44 chapters from a multitude of authors providing the first comprehensive academic treatment of the problem of freedom on the space frontier. Within a year after publication of the final book, the trilogy had surpassed over 10,000 individual chapter downloads.

As these meetings lasted for just 2 days each year, they made a minimum disruption to core funded activities, yet they took our science objectives of studying life in extremes into a highly productive and interesting interaction with political philosophy.

## 8. Education and Social Reform

When UKCA was established, we sought to contribute to astrobiology education. Our motivation for doing this was to use UKCA to achieve the improvement of astrobiology education locally and internationally. These initiatives have provided PhD students and postdoctoral scientists at UKCA with an opportunity to engage in public outreach. Here, we summarize some specific projects, lessons we have learned, and how we integrated these into center activities.

### 8.1. University education

The use of astrobiology in university education is not new, and several courses have been attempted in different institutions and their findings discussed previously (Danly, 2004; Tang, 2005; Dartnell and Burchell, 2009; Foster and Drew, 2009). At the University of Edinburgh, we launched a first-year undergraduate elective course in astrobiology, which began in the 2013/14 academic year. In its first year, the course attracted 110 students. By the 2015/16 academic year, the course attracted 200 students (at which it was capped) from 11 different schools across the university that cut across the Colleges of Science and Engineering; Medicine and Veterinary Science; and Arts, Humanities, and Social Sciences. The course is well received. The formal feedback process within the university (comprising written surveys at the end of every course) suggests that one of the most exciting aspects of the course that engages undergraduates is the cross-link between sciences studied in the context of questions that remain unanswered, such as the origin of life, and therefore capture high levels of curiosity.

As at other universities, the astrobiology course at Edinburgh can be considered as a grounding in general science aligned to specific questions related to the origin and evolution of life. Its 33 lectures cover the following themes: the structure of living things, the origin of life, the history of life on Earth, introduction to the Solar System, stellar evolution and planet formation, astrochemistry and organics in space, the habitability of planets and Earth as a location for life, exoplanet formation and habitability, and SETI. During the course, some of the research at UKCA is used to illustrate ideas in astrobiology, bringing students into contact with the research environment and bringing our science and education objectives together.

One of the most powerful phenomena we have observed in student feedback is that biologists who dropped physics at high school, for example, suddenly find a new interest in physics. Conversely, physicists who dropped biology at school find a new enthusiasm in the subject when it is considered from an astronomical perspective. In general, the interdisciplinary nature of astrobiology provides an opportunity for students to cross from their chosen degree course into other fields.

The astrobiology course (and the presence of UKCA) had knock-on effects in the undergraduate community. In 2013, the University of Edinburgh became host to the United Kingdom's first undergraduate astrobiology society, which in 2016 had just over 50 members. This type of activity illustrates how a center can use its activity to contribute to the undergraduate social environment.

The teaching of this course led to the writing of an undergraduate textbook with Wiley-Blackwell in 2015 entitled *Astrobiology: Understanding Life in the Universe* (Cockell, 2015c). The textbook was written with the objective of providing a text developed from a university-level course that could be used in other universities. In addition to the book, 22 sets of slides were provided covering each of its chapters with each set of slides constituting a 50 min lecture, allowing anyone to establish an astrobiology course at a university. This entire slide set was translated into Spanish. These slide sets can be obtained from Wiley or the lead author.

We have found other ways to integrate UKCA's work into education. Research activities at UKCA have been linked with individual and group-based student projects. These have included master's students from the University's School of Physics and Astronomy, the School of Geosciences, and the School of Biological Sciences. About three master's students have been involved each year. One of the key features of the projects was to provide students with an opportunity to become acquainted with fields beyond their immediate area of experience (*e.g.,* microbial ecology for physics students) and to participate in the publication of peer-reviewed papers. Despite these projects requiring the students to work on topics that may extend beyond their chosen degree course, they have been a constant source of positive feedback.

To summarize, we strongly recommend that an astrobiology center, particularly if it is located at a university, engage in undergraduate teaching. The specific contributions that we found a center can make are (1) bringing science undergraduates up to a certain standard of knowledge across all sciences, (2) teaching students that good scientific questions often do not conform to convenient disciplinary definitions, (3) re-igniting enthusiasm for science subjects that undergraduates may have dropped earlier at school, (4) providing lab-based training, (5) engaging social scientists and humanities students in the sciences that underpin astrobiology, (6) generally widening students' perceptions of the scope of science, (7) involving master's students and other undergraduate groups in primary research.

### 8.2. Primary and high school education—the Astrobiology Academy

We recognized from an early stage that the astrobiology expertise we had gathered could be used to further primary and high school education. The origin and evolution of life and the search for life on other planetary bodies are of intrinsic interest to a large number of school pupils, yet these questions are underpinned by much of the fundamental science that forms part of science curricula anywhere in the world.

In 2013, we formed the Astrobiology Academy (http://www.astrobiologyacademy.org) to take astrobiology to secondary schools. In the first iteration (July 2013), we hosted 20 high school students at the University of Edinburgh who spent a week listening to astrobiology lectures, interspersed with discussion groups and a live question-and-answer session with British astronaut Tim Peake. The main focus of the week was working toward the last day, on which students put on a public outreach event on astrobiology led by them. We found the results spectacularly successful, and the ingenuity they expressed was impressive. For example, molecular concentration during the origin of life was demonstrated by using Lego bricks in water, gelatin, and sand to show the challenges of bringing molecules together. Life detection was demonstrated by using ink squeezed from a UV marker onto rocks, with the public required to use a UV lamp to find it—a demonstration of fluorescent life detection.

In July 2014, we ran the Astrobiology Academy again for secondary school students with similar success. In both 2013 and 2014, the participation of US graduates was supported by the NAI.

A limitation of this format was always the logistically intensive work and the impact on a small cross section of students. Twenty students had a remarkable time, but the impact on UK secondary school education more widely can only be limited.

**FIG. 10.**
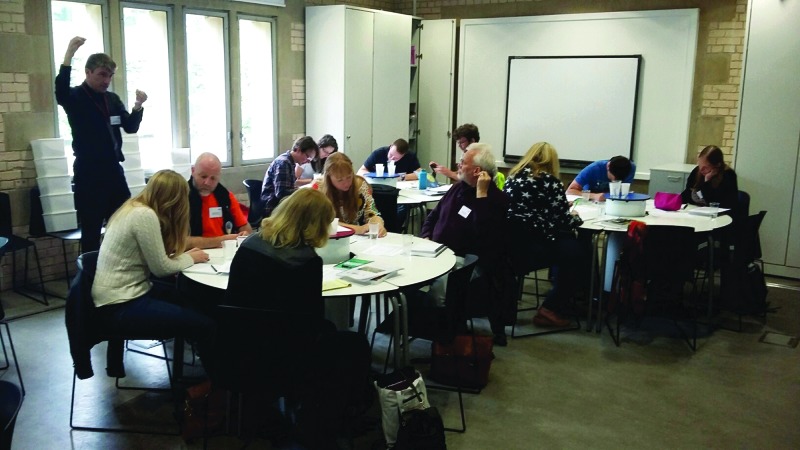
The Astrobiology Academy brings school teachers across the United Kingdom together to learn astrobiology and develop new classroom and curriculum materials that incorporate astrobiology.

Recognizing the limited impact in focusing on students, in 2014, immediately following the student event, we ran a 3-day continuing professional development (CPD) course for 15 secondary school teachers from across the United Kingdom to engage them in astrobiology (Fig. 10). During the event, they received astrobiology lectures and took part in high school activities involving exoplanet detection and life in extremes that could be implemented in the classroom. We would note that the 2 years we ran the academy with secondary school students were useful in acquiring an understanding of what fires the imagination of school students, which subsequently helped facilitate planning the schedule of the teacher training initiative.

The main focus of the teacher training event was to write lesson plans for primary and secondary schools by taking existing curriculum subjects and transforming them into astrobiology. For example, a conservative and quite standard lesson plan on photosynthesis can be turned into a discussion on the extremes of the surface of Mars (*e.g.,* lack of liquid water because of the low temperature and atmospheric pressure) and how they would affect photosynthesis. Thus, standard subjects in the curriculum were made more engaging by taking them into unfamiliar and alien environments. Fourteen astrobiology lesson plans were written, each of which was split into modular activities that could be mixed and matched, and aligned with UK science curriculum standards and objectives. They were further developed with teachers after the academy.

One way in which we have linked the academy to our science objectives and integrated it into center activity is to get PhD students in UKCA to provide short talks to teachers on their research. This has been well received since it provides teachers with cutting-edge research that they can incorporate into their teaching material and provides them with confidence that they are up-to-date in a variety of areas of science. These talks are interspersed with discussion with teachers on how to incorporate active astrobiology research into teaching. Laboratory tours were used to familiarize them with the practical aspects of astrobiology research.

Since then the academy has continued annually to focus on the development of curriculum materials and putting together our existing lesson plans into full astrobiology units with a coherent set of interconnected lessons that could be taught in schools over the course of a year.

The result of this work is the *Astrobiology in the Classroom* lesson plan and unit resource that is downloadable from the academy website. Our lesson plans were uploaded onto the TES Resource website, a key resource for science teachers, and within a year they had been downloaded over 6000 times. In 2016, we developed the *Astrobiology in a Box* resource, a simple set of five ready-to-use activities in a box that can be used in lessons and teach fundamental concepts in physics and biology. These are now being used in primary schools across Scotland by local authorities. The list of box contents and activities is downloadable from the website.

### 8.3. Public education

Astrobiology lends itself to public science education. Its component disciplines, which cover most scientific fields, come together in questions of broad interest to the general public.

As part of an initiative launched by the University of Edinburgh at the end of 2011 to take part in the newly emerging activity of massive open online courses (MOOCs) (Baggaley, 2013), UKCA was asked to put together such a course for public engagement, one of six courses assembled by the university.

We constructed a 5-week program offered as Astrobiology and the Search for Extraterrestrial Life by Coursera, a free publicly available platform for online courses (https://www.coursera.org/learn/astrobiology). Globally, it was the first MOOC to be launched in astrobiology. The course became available in 2012. It comprised five modules as follows:

(1) The history of astrobiology, the structure of life, and the origin of life;
(2) The formation of the Solar System, Earth, and process of biological evolution, covering the evidence for life in the rock record, the Great Oxidation Event, and the rise of multicellularity;
(3) The search for life on Mars and the habitability of icy moons;
(4) The search for exoplanets and potential biosignatures;
(5) The possibility of intelligent life and the sociological implications of astrobiology.

The course was constructed of 30 lectures of approximately 10–20 min each. The course was assessed by quizzes at the end of each weekly module. We integrated this into center activities by giving PhD students and postdoctoral students the opportunity to help develop the course material and subsequently oversee the discussion forums, offering them the opportunity to gain expertise in distance learning initiatives.

Initially, the course was offered as a once-yearly enrollment, but in 2014 it transitioned to “on-demand,” allowing anyone at any time to take the course. By September 2016, over 120,000 people from 83 countries had taken part in the course. Between 2014 and the start of 2017, 3653 of the 41,350 students that took part in parts of the course completed the whole course (this 8.8% retention rate until course completion compares favorably to typical Coursera rates of about 6%). This reflects the large number of people who cherry-pick sections of the course to engage in, but nevertheless the substantial number that complete all the lectures and quizzes. The course attracted a wide range of ages, from students less than 18 years old to those greater than 65. The peak age bin was 25–34 (Woodgate *et al.,* 2015).

The Coursera offering also reached into social media. Following the first iteration of the course, a group of students formed the Virtual Astrobiology Society on Facebook, a forum for disseminating astrobiology news and information. Its membership exceeded 10,000 in 2016.

The MOOC demonstrated the vast public interest in astrobiology. We recommend that an astrobiology center leverage its research interests toward public education and develop such a strand to its work. In our case, the MOOC involved a large amount of work to put the course together, but once it was complete, it required very little work to sustain. Most of the effort involved overseeing and interacting with learners through the discussion forums.

We have since used the MOOC lectures for undergraduate education and provided the lectures to participants in the Astrobiology Academy as an introductory resource material, showing how education activities within a center can cross-fertilize other initiatives.

One essential requirement in any public education is that the workload required year-on-year be estimated before embarking on these ventures. The University of Edinburgh placed significant resources into the MOOC enterprise, establishing a team responsible for the courses offered by the university, ensuring that UKCA had adequate logistics and administrative support throughout. We recommend that any plan to engage in such an enterprise consider this requirement.

### 8.4. Prison education and social reform

**FIG. 11.**
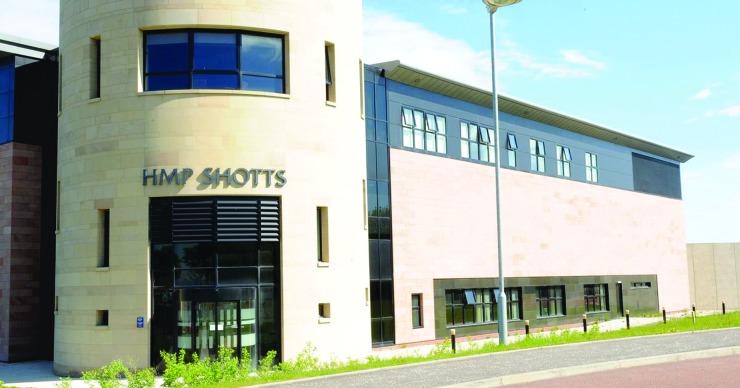
Her Majesty's Prison (HMP) Shotts in Scotland, a maximum-security prison for long-term male offenders. The prison is one location taking part in the UKCA's Life Beyond program using astrobiology for education and reform in the prison population.

In 2016, UKCA began an initiative in collaboration with the Scottish Prison Service, entitled Life Beyond (Fig. 11). The program was motivated by the expertise UKCA had acquired in astrobiology education and a sense that this expertise could be usefully applied to other groups of the public who do not normally get these opportunities to learn.

The purpose of this program is to take astrobiology into prisons and thereby enhance science education. It is focused on engaging offenders (particularly long-term offenders) in the major challenges that humanity faces in living sustainably on a planet and the major opportunities that our civilization has in developing a space-faring society with all the possibilities that entails, such as creating a multiplanet species.

In addition, Life Beyond can contribute to civic responsibility. By involving offenders in thinking about the large-scale challenges facing our society, they can be encouraged to see that criminal activity merely slows progress and degrades the quality of life in a society that already has huge challenges to address and yet also has immense potential. In this way, inmates see that they are part of that future; and the major questions of astrobiology, particularly those focusing on the human future, act as a foundation to stir the imagination of the prison population and their sense of belonging to a civilization.

The program began with a pilot phase at four prisons in Scotland that involved an astrobiology lecture followed by an inmate-led discussion group about living on Mars. Following the pilot phase, feedback from the ∼100 inmates that took part was gathered by the Scottish Prison Service on what aspects of astrobiology they found most interesting. This pilot phase has led to the development of a 4-week course. The Life Beyond course focuses on a design study for a station on Mars, involving inmates in science, technology, creative writing, and a diversity of other skills. The station designs and associated creative products are to be published, providing the inmates with a tangible output to their efforts (Cockell *et al.,* 2018).

Life Beyond taps into our astrobiology expertise and involves PhD students from UKCA in delivering the course and providing them with the opportunity to teach in prisons and widen their experience in science teaching in general. Both our MOOC materials and astrobiology textbook are also used to facilitate teaching, again providing an example of economies of scale achieved in education activities.

### 8.5. Astronaut training

The UK Centre for Astrobiology is a participant in the ESA Pangaea program, an ESA initiative to train astronauts in skills necessary for planetary exploration. In early 2017, UKCA hosted ESA astronauts Matthias Maurer and Pedro Duque for a 2-day astrobiology and field training event in which they learned about basic concepts in astrobiology relevant to the study of rocky environments and the search for life. They practiced aseptic methods in UKCA labs and sterile sample collection methods in the field on Arthur's Seat, Edinburgh. This education effort draws on our MOOC materials, university lecture materials, analog activity, and laboratory research in UKCA, showing how integrated science and education activity in an astrobiology center can be used to prepare human explorers who take part in missions beyond Earth.

## 9. Other Overarching Lessons Learned

In addition to the lessons learned and discussed above, here we highlight four particular large-scale lessons.

### 9.1. To build or not to build?

Should astrobiology centers aspire to construct buildings? At UKCA, we concluded that this ambition may not even be in the interests of astrobiology. Astrobiology is a huge field with many intersecting disciplines, each of which can occupy a whole university department. Achieving a critical mass to make an entire astrobiology center within one building that covers many areas of the field requires considerable resources. The Spanish Centre for Astrobiology has achieved success in garnering this level of activity, but this is unusual, and long-term sustainability is a serious challenge.

One advantage we have found of a virtual group is the ability to fluidly involve people from different fields and departments within and outside an organization as projects and ideas develop. In the case of UKCA, this has minimized the overburden of estates and logistics and has minimized the logistical commitment, allowing us to focus on science, technology, and education activities. The one exception is the construction of the underground astrobiology laboratory. However, this facility is part of a larger underground facility.

Science is often segmented into disciplinary compartments for logistical and administrative reasons. While this may be convenient, it can have the effect of segmenting scientists in their intellectual connections. Astrobiology is just one area of science that, in reaching across disciplinary boundaries and creating a presence in many different departments, has the potential to encourage cross-fertilization of ideas across traditionally separated disciplines. This is most easily done by being a virtual group that can involve people from different departments and universities.

### 9.2. A jack-of-all-trades or a master of one?

Astrobiology covers a vast diversity of areas from the origins of life to the activity of humans beyond Earth. We found that it was essential to focus on one particular area. In the case of UKCA, we focused on life in extreme environments and habitability.

The extent to which a center can expand into the numerous areas of astrobiology obviously depends on the resources it has access to and volunteers or contributing scientists. We modulated our focus to ensure that we can achieve a credible level of output of peer-reviewed papers in a well-focused area. We recommend that anyone setting up an astrobiology center begin by clearly defining the area (or areas) in which it will initially operate rather than attempting to cover all areas of astrobiology. These choices will be determined by the expertise of the founders of the center and/or the institutional expertise it has access to that will form the core of the group's activities.

### 9.3. National or not national?

When we established UKCA, we engaged in a discussion on our plans and ideas with the NAI in advance of developing an affiliate status link with the NAI. Similar to the Spanish and Australian Centres for Astrobiology, NASA was keen to establish a link with a national “node” that could widen the net of national astrobiology groups interacting with one another.

The question of whether a center should assume a national status hinges on the extent of astrobiology activity in a given country. In a nation that has little or nascent astrobiology activity or a small population, a national center can consolidate critical mass and bring governmental and other institutional attention to the science. Indeed, this was the rationale for many of the existing national astrobiology centers. Even where there is existing astrobiology activity, a center that acts as a national node has its international merits.

On balance, we considered a UK Centre for Astrobiology a worthwhile enterprise given the existence of successful centers in other nations and the desire to draw upon the successful astrobiology activity ongoing within the United States.

Establishing a national center brings with it unique challenges. We have found it extremely challenging to contribute to a nationwide effort to pull together all astrobiologists, a task that is probably better left to community societies (in our case, the Astrobiology Society of Britain). As running a center requires a great deal of local administrative work coordinating many activities, any center naturally tends to become local in its functioning. Nevertheless, we have found our infrastructure research projects and community efforts such as the Building Habitable Worlds workshop to be effective ways to engage the community more widely.

### 9.4. Using research for technology and education

Astrobiology is not unique in its potential for transfer into other nonscience activities, but the interdisciplinary scope of its questions and its reliance on space missions and the exploration of other planetary bodies does produce a particularly rich seam of possibilities for linking science to technology development and education. In our case, the development of a deep subsurface analog program, a MOOC, and the development of the Astrobiology Academy are just examples of how a core science activity can be used to provide sustenance to allied activities. One challenge we have found is ensuring that these other activities do not erode the focus of early career researchers on getting science done. Ensuring that this does not occur is partly a function of personnel management, but it is also important to create well-defined technology and education projects whose limits are carefully circumscribed to make them useful contributions, but not a burden. For example, in the case of the Astrobiology Academy there have been many potential new directions we could have taken this in, including astrobiology training courses at individual schools around the country. However, we chose to focus on fixed training events directed at astrobiology lesson plans and unit development to maximize impact whilst controlling the extent of time commitment required by those in UKCA.

## 10. The Future

The UK Centre for Astrobiology will operate for another 5 years, giving it a total of 10 years of contribution. At that stage, we will decide on one of four paths: (1) Continue for another 5 years, (2) Hand over UKCA to another interested institution, (3) Morph UKCA into something different or change its focus or remit, (4) End the enterprise. None of these necessarily constitute more or less negative outcomes.

The first 5 years of UKCA (Phase One) involved the innovation of initiatives and establishment of coherent lines of activity in research, teaching, and education. For the next 5 years (Phase Two), we will focus on continuing the existing initiatives described in this paper, achieving greater depth in our chosen research, technology, and education contributions. Phase Two will consist of driving each of these lines of activity to 5–10 years of consistent and conclusive fruitful outputs.

## 11. Conclusions

We conclude by observing that establishing centers and institutions in astrobiology is enormously rewarding and beneficial. Astrobiology lends itself not merely to the pursuit of good scientific questions but to providing a basis for general science education from primary school to university level curricula to instruction for the incarcerated. Its compelling questions make astrobiology a powerful way to achieve public education in science. In linking science to diverse planetary environments on Earth and elsewhere, astrobiology can also yield advances in technology development. Thus, every effort in establishing new institutions that advance this work is to be encouraged. We hope that the experiences described here will be helpful to others who aspire to launch new institutional efforts to advance this science. This paper may even provide some useful advice for individuals considering establishing a center in other fields of science.

## Abbreviations Used

BASALT: Biologic Analog Science Associated with Lava Terrains
BISAL: Boulby International Subsurface Astrobiology Laboratory
EANA: European Astrobiology Network Association
EU: European Union
EVA: extravehicular activity
GeoRepNet: Geological Repositories Network
MASE: Mars Analogues for Space Exploration
MINAR: Mine Analogue Research
MOOCs: massive open online courses
NAI: NASA Astrobiology Institute
STFC: Science and Technology Facilities Council
UKCA: UK Centre for Astrobiology

## References

[B1] Baggaley J. (2013) MOOC rampant. Distance Education 34:368–378

[B2] *Beblo-Vranesevic K., Bohmeier M., Perras A.K., Schwendner P., Rabbow E., Moissl-Eichinger C., Cockell C.S., Pukall R., Vannier P., Marteinsson V.T., Monaghan E.P., Ehrenfreund P., Garvia-Descalzo L., Gómez F., Malki M., Amils R., Gaboyer F., Westall F., Cabezas O., Walter N., and Rettberg R. (2017) The responses of an anaerobic microorganism, *Yersinia intermedia* MASE-LG-1, to individual and combined simulated martian stresses. PLoS One 12, doi:10.1371/journal.pone.0185178 PMC5656303 29069099

[B3] Boston P.J., Ivanov M.V., and McKay C.P. (1992) On the possibility of chemosynthetic ecosystems in subsurface habitats on Mars. Icarus 95:300–308 11539823 10.1016/0019-1035(92)90045-9

[B4] Bowler S. (2013) From outer space to mining. Astron Astrophys 54:3.25–3.27

[B5] *Brown S.P., Mead A.J., Forgan D.H., Raven J.A., and Cockell C.S. (2014) Photosynthetic potential of planets in 3:2 spin-orbit resonances. International Journal of Astrobiology 13:279–289

[B6] *Bryce C., Horneck G., Rabbow E., Edwards H.G.M., and Cockell C.S. (2015) Impact shocked rocks as protective habitats on an anoxic early Earth. International Journal of Astrobiology 14:115–122

[B7] *Bryce C., LeBihan T., Martin S.F., Harrison J.P., Bush T., Spears B., Moore A., Leys N., Byloos B., and Cockell C.S. (2016) Rock geochemistry induces stress and starvation responses in the bacterial proteome. Environ Microbiol 18:1110–11121 26470852 10.1111/1462-2920.13093

[B8] Chela-Flores J. (2011) When astrobiology meets philosophy. In The Science of Astrobiology, Cellular Origin, Life in Extreme Habitats and Astrobiology Vol. 20, Springer, Dordrecht, The Netherlands, pp 247–256

[B9] Cockell C.S. (2010) Geomicrobiology beyond Earth—microbe-mineral interactions in space exploration and settlement. Trends Microbiol 18:308–314 20381355 10.1016/j.tim.2010.03.005

[B10] *Cockell C.S. (2014a) Trajectories of martian habitability. Astrobiology 14:182–203 24506485 10.1089/ast.2013.1106 PMC3929387

[B11] *Cockell C.S. (2014b) Habitable worlds with no signs of life. Philos Trans A Math Phys Eng Sci 372, doi:10.1098/rsta.2013.0082 PMC3982426 24664917

[B12] *Cockell C.S. (2014c, 5 29) The 500-year microbiology experiment. Microbiology Today 5:95

[B13] *Cockell C.S. (2014d) Types of habitat in the Universe. International Journal of Astrobiology 13:158–164

[B14] *Cockell C.S., editor. (2015a) The Meaning of Liberty Beyond Earth, Springer, Heidelberg

[B15] *Cockell C.S., editor. (2015b) Human Governance Beyond Earth: Implications for Freedom, Springer, Heidelberg

[B16] *Cockell C.S. (2015c) Astrobiology: Understanding Life in the Universe, Wiley-Blackwell, Chichester, UK

[B17] *Cockell C.S., editor. (2016) Dissent, Revolution and Liberty Beyond Earth, Springer, Heidelberg

[B18] *Cockell C.S. and Nixon S.L. (2013) The boundaries of life. In Astrochemistry and Astrobiology, edited by Smith I.W.M., Cockell C.S., and Leach S., Springer, Heidelberg, pp 211–242

[B19] Cockell C.S., Rettberg P., Rabbow E., and Olsson-Francis K. (2011) Exposure of phototrophs to 548 days in low Earth orbit: microbial selection pressures in outer space and on early Earth. ISME J 5:1671–1682 21593797 10.1038/ismej.2011.46 PMC3176519

[B20] *Cockell C.S., Payler S., Paling S., and McLuckie D. (2013) Boulby International Subsurface Astrobiology Laboratory. Astronomy and Geophysics 54:2.25–2.27

[B21] *Cockell C.S., Bush T., Bryce C., Direito S., Fox-Powell M., Harrison J.P., Lammer H., Landenmark H., Martin-Torres J., Nicholson N., Noack L., O'Malley-James J., Payler S.J., Rushby A., Samuels T., Schwendner P., Wadsworth J., and Zorzano M.P. (2015a) Habitability: a review. Astrobiology 16:89–117 10.1089/ast.2015.1295 26741054

[B22] *Cockell C.S., Cousins C., Wilkinson P.T., Olsson-Francis K., and Rositis B. (2015b) Are thermophilic microorganisms active in cold environments? International Journal of Astrobiology 14:457–463

[B23] *Cockell C.S., Samuels T., Sirks E., Mayer M., Friswell I., Nicholson T., Schröder A., Nagler K., Raguse M., Rettberg P., and Moeller R. (2015c) A 500-year experiment. Astronomy and Geophysics 56:1.28–1.29

[B24] *Cockell C.S., Schwendner P., Perras A., Rettberg P., Beblo-Vranesevic K., Bohmeier M., Rabbow E., Moissl-Eichinger C., Wink L., Marteinsson V., Vannier P., Gomez F., Garcia-Descalzo L., Ehrenfreund P., Monaghan E.P., Westall F., Gaboyer F., Amils R., Malki M., Pukall R., Cabezas P., and Walter N. (2017) Anaerobic microorganisms in astrobiological analogue environments: from field site to culture collection. International Journal of Astrobiology, in press. doi:10.1017/S1473550417000246

[B25] *Cockell C.S., Fosado Y., Hitchen J., Landenmark H., Perera L., Schwendner P., and Vissers T. (2018) Life Beyond–A program to use astrobiology to teach science and advance space exploration through prisons. Journal of Correctional Education, in press

[B26] Coombs C.R. and Hawke B.R. (1992) A search for intact lava tubes on the Moon: possible lunar base habitats. In The Second Conference on Lunar Bases and Space Activities of the 21^st^ Century, NASA Conf. Publ., NASA CP-3166, 1:219–229

[B27] *Cousins C.R. (2015) Volcanogenic fluvial-lacustrine environments in Iceland and their implication for identifying past habitability on Mars. Life 5:568–586 25692905 10.3390/life5010568 PMC4390869

[B28] *Cousins C.R., Crawford I.A., Carrivick J.L., Gunn M., Harris J., Kee T.P., Karlsson M., Carmody L., Cockell C.S., Herschy B., and Joy K.H. (2013) Glaciovolcanic hydrothermal environments in Iceland and implications for their detection on Mars. Journal of Volcanology and Geothermal Research 256:61–77

[B29] *Cousins C.R., Cockell C.S., and the Geobiology in Space Exploration Topical Team. (2015) An ESA roadmap for geobiology in space exploration. Acta Astronaut 118:286–295

[B30] Cushing G.E., Titus T.N., Wynne J.J., and Christensen P.R. (2007) THEMIS observes possible cave skylights on Mars. Geophys Res Lett 34:L17201

[B31] Danly L. (2004) Life in the Universe: a multidisciplinary science curriculum for undergraduate honors students. Bulletin of the American Astronomical Society 36:676

[B32] Dartnell L.R. and Burchell M.J. (2009) Survey on astrobiology research and teaching activities within the United Kingdom. Astrobiology 9:717–730 19845444 10.1089/ast.2009.0348

[B33] De Angelis S.H. (2017) Earth science at the UK's deepest laboratory. Geology Today 33:132–137

[B34] Des Marais D.J., Allamandola L.J., Benner S.A., Boss A.P., Deamer D., Falkowski P.G., Farmer J.D., Hedges S.B., Jakosky B.M., Knoll A.H., Liskowsky D.R., Meadows V.S., Meyer M.A., Pilcher C.B., Nealson K.H., Spormann A.M., Trent J.D., Turner W.W., Woolf N.J., and Yorke H.W. (2003) The NASA Astrobiology Roadmap. Astrobiology 3:219–235 14577870 10.1089/153110703769016299

[B35] *Direito S., Clark S., Cousins C., Fujita Y., Gluyas J., Harley S., Holmes R.J., Hutchinson I., Kudryavtsev V.A., Lloyd J., Main I.G., Nayloe M., Payler S., Smith N., Spooner N.J.C., Telfer S., Thompson L.F., Wouters K., Wragg J., and Cockell C.S. (2015) Geological repositories: scientific priorities and potential high technology transfer from the space sector. Mineralogical Magazine 11, doi:10.1180/minmag.2015.079.6

[B36] Fernández-Remolar D.C., Prieto-Ballesteros O., Rodriguez N., Gómez F., Amils R., Gómez-Elvira J., and Stoker C.R. (2008) Underground habitats in the Río Tinto basin: a model for subsurface life habitats on Mars. Astrobiology 8:1023–1047 19105758 10.1089/ast.2006.0104

[B37] *Forgan D.H., Mead A., Cockell C.S., and Raven J.A. (2015) Surface flux patterns on planets in circumbinary systems and potential for photosynthesis. International Journal of Astrobiology 14:465–478

[B38] *Forgan D.H., Dayal P., Cockell C.S., and Libeskind N. (2017) Evaluating galactic habitability using high-resolution cosmological simulations of galaxy formation. International Journal of Astrobiology 16:60–73

[B39] Foster J.S. and Drew J.C. (2009) Astrobiology undergraduate education: students' knowledge and perceptions of the field. Astrobiology 9:325–333 19355819 10.1089/ast.2007.0221

[B40] *Fox-Powell M.G., Hallsworth J.E., Cousins C.R., and Cockell C.S. (2016) Ionic strength is a barrier to the habitability of Mars. Astrobiology 16:427–442 27213516 10.1089/ast.2015.1432

[B41] *Freeman K., Harrison J.P., Dobinson L., Cockell C.S., McKenzie R., Wylie D., and Nixon S.L. (2016) Mapping limits to life on Earth. Astronomy and Geophysics 57:2.15–2.17

[B42] *Gaboyer F., Le Milbeau C., Bohmeier M., Schwendner P., Vannier P., Beblo-Vranesevic K., Rabbow E., Foucher F., Gautret P., Guégan R., Richard A., Sauldubois A., Richmann P., Perras A.K., Moissl-Eichinger C., Cockell C.S., Rettberg P., Marteinsson V., Monaghan E., Ehrenfreund P., Garcia-Descalzo L., Gomez F., Malki M., Amils R., Cabezas P.O., Walter N., and Westall F. (2017) Mineralisation and preservation of an extremotolerant bacterium isolated from an early Mars analog environment. Sci Rep 7, doi:10.1038/s41598-017-08929-4 PMC5562696 28821776

[B43] Grotzinger J.P., Sumner D.Y., Kah L.C., Stack K., Gupta S., Edgar L., Rubin D., Lewis K., Schieber J., Mangold N., Milliken R., Conrad P.G., Des Marais D., Farmer J., Siebach K., Calef F., Hurowitz J., McLennan S.M., Ming D., Vaniman D., Crisp J., Vasavada A., Edgett K.S., Malin M., Blake D., Geliert R., Mahaffy P., Wiens R.C., Maurice S., Grant J.A., Wilson S., Anderson R.C., Beegle L., Arvidson R., Hallet B., Sletten R.S., Rice M., Bell J., Griffes J., Ehlmann B., Anderson R.B., Bristow T.F., Dietrich W.E., Dromart G., Eigenbrode J., Fraeman A., Hardgrove C., Herkenhoff K., Jandura L., Kocurek G., Lee S., Leshin L.A., Leveille R., Limonadi D., Maki J., McCloskey S., Meyer M., Minitti M., Newsom H., Oehler D., Okon A., Palucis M., Parker T., Rowland S., Schmidt M., Squyres S., Steele A., Stolper E., Summons R., Treiman A., Williams R., Yingst A., and the MSL Science Team. (2014) A habitable fluvio-lacustrine environment at Yellowknife Bay, Gale Crater, Mars. Science 343, doi:10.1126/science.1242777 24324272

[B44] Hand K.P. and Chyba C.F. (2007) Empirical constraints on the salinity of the europan ocean and implications for a thin ice shell. Icarus 189:424–438

[B45] *Harris J.K., Cousins C.R., Gunn M., Grindrod P.M., Barnes D.P., Crawford I.A., Cross R., and Coates A.J. (2015) Remote detection of past habitability at Mars-analogue hydrothermal alteration terrains using an ExoMars Panoramic Camera emulator. Icarus 252:284–300

[B46] *Harrison J.P., Gheeraert N., Tsigelnitskiy D., and Cockell C.S. (2013) The limits for life under multiple extremes. Trends Microbiol 21:204–212 23453124 10.1016/j.tim.2013.01.006

[B47] *Harrison J.P., Hallsworth J.E., and Cockell C.S. (2015a) Reduction of the temperature sensitivity of *Halomonas hydrothermalis* by iron starvation combined with microaerobic conditions. Appl Environ Microbiol 81:2156–2162 25595757 10.1128/AEM.03639-14 PMC4345356

[B48] *Harrison J.P., Dobinson L., Freeman K., McKenzie R., Wyllie D., Nixon S.L., and Cockell C.S. (2015b) Aerobically respiring prokaryotic strains exhibit a broader temperature–pH–salinity space for cell division than anaerobically respiring and fermentative strains. J R Soc Interface 12, doi:10.1098/rsif.2015.0658 PMC4614477 26354829

[B49] *Harrison J.P., Aggarwal S.D., and Cockell C.S. (2016) Salinity influences the response of *Halomonas hydrothermalis* to artificial fossilization by evaporative silicification. Geomicrobiol J 33:377–386

[B50] *Harrison J.P., Angel R., and Cockell C.S. (2017) Astrobiology as a framework for investigating antibiotic susceptibility: a study of *Halomonas hydrothermalis*. J R Soc Interface 14, doi:10.1098/rsif.2016.0942 PMC5310740 28123098

[B51] Hobbes T. (1651) Leviathan, Andrew Crooke, London

[B52] *Josset J.-L., Westall F., Hofmann B.A., Spray J., Cockell C.S., Kempe S., Griffiths A.D., Cristina de Sanctis M., Colangeli L., Koschny D., Föllmi K., Verrecchia E., Diamond L., Josset M., Javaux E.J., Esposito F., Gunn M., Souchon A.L., Bontognali T., Korablev O., Erkman S., Paar G., Ulamec S., Foucher F., Martin P., Verhaeghe A., Tanevski M., and Vago J.L. (2017) The Close-Up Imager onboard the ESA ExoMars rover: objectives, description, operations, and science validation activities. Astrobiology 17:595–611 28731819 10.1089/ast.2016.1546

[B53] Kallmeyer J. and Wagner D. (2014) Microbial Life of the Deep Biosphere, De Gruyter, Germany

[B54] *Kelly L.C., Cockell C.S., Thorsteinsson T., Marteinsson V., and Stevenson J. (2014) Pioneer microbial communities of the Fimmvörðuháls Lava Flow, Eyjafjallajökull, Iceland. Microb Ecol 68:504–518 24863128 10.1007/s00248-014-0432-3

[B55] Khurana K.K., Kivelson M.G., Stevenson D.J., Schubert G., Russell C.T., Walker R.J., and Polanskey C. (1998) Induced magnetic fields as evidence for subsurface oceans in Europa and Callisto. Nature 395:777–780 9796812 10.1038/27394

[B56] *Landenmark H.K.E., Forgan D.H., and Cockell C.S. (2015) An estimate of the total DNA in the biosphere. PLoS Biol 13, doi:10.1371/journal.pbio.1002168 PMC4466264 26066900

[B57] Locke J. (1689) Two Treatises of Government, Awnsham Churchill, London

[B58] Lorenz R.D., Stiles B.W., Kirk R.L., Allison M.D., del Marmo P.P., Less L., Lunine J.L., Ostro S.J., and Hensley S. (2008) Titan's rotation reveals an internal ocean and changing zonal winds. Science 319:1649–1651 18356521 10.1126/science.1151639

[B59] *Loudon C.-M., Nicholson N., Finster K., Leys N., Byloos B., Van Houdt R., Rettberg P., Moeller R., Fuchs F., Demets R., Krause J., Vukich M., Mariani A., and Cockell C.S. (2018) BioRock: new experiments and hardware to investigate microbe–mineral interactions in space. International Journal of Astrobiology, in press, doi:10.1017/S1473550417000234

[B60] *Martin D. and Cockell C.S. (2015) PELS (Planetary Environmental Liquid Simulator): a new type of simulation facility to study extraterrestrial aqueous environments. Astrobiology 15:111–118 25651097 10.1089/ast.2014.1240

[B61] *Martin-Torres F.J., Zorzano M.P., Valentin-Serrano P., Harri A.M., Genzer M., Kemppinen O., Rivera-Valentin E.G., Jun I., Wray J., Madsen M.B., Goetz W., McEwan A.S., Hardgrove C., Renno N., Chevrier V.F., Mischna M., Navarro-Gonzalez R., Martinez-Frias J., Conrad P., McConnochie T., Cockell C., Berger G., Vasavada A.R., Sumner D., and Vaniman D. (2015) Transient liquid water and water activity at Gale Crater on Mars. Nat Geosci 8:357–361

[B62] *Moissl-Eichinger C., Cockell C.S., and Rettberg P. (2016) Venturing into new realms? Microorganisms in space. FEMS Microbiol Rev 40:722–737 27354346 10.1093/femsre/fuw015

[B63] *Morgan J.V., Gulick S.P.S., Bralower T., Chenot E., Christeson G., Claeys P., Cockell C.S., Collins G.S., Coolen M.J.L., Ferriere L., Gebhardt C., Goto K., Jones H., Kring D.A., Le Ber E., Lofi J., Long X., Lowery C., Mellett C., Ocampo-Torres R., Osinski G.R., Perez-Cruz L., Pickersgill A., Poelchau M., Rae A., Rasmussen C., Rebolledo-Vieyra M., Riller U., Sato H., Schmitt D.R., Smit J., Tikoo S., Tomioka N., Urrutia-Fucugauchi J., Whalen M., Wittmann A., Yamaguchi K.E., and Zylberman W. (2016) The formation of peak rings in large impact craters. Science 354:878–882 27856906 10.1126/science.aah6561

[B64] *Nixon S.L. and Cockell C.S. (2015) Nonproteinogenic D-amino acids at millimolar concentrations are a toxin for anaerobic microorganisms relevant to early Earth and other anoxic planets. Astrobiology 15:238–246 25695622 10.1089/ast.2014.1252

[B65] *Nixon S.L., Cockell C.S., and Tranter M. (2012) Limitations to a microbial iron cycle on Mars. Planet Space Sci 72:116–128

[B66] *Nixon S.L., Cousins C.R., and Cockell C.S. (2013) Plausible microbial metabolisms on Mars. Astronomy and Geophysics 54:1.13–1.16

[B67] *Nixon S.L., Telling J.P., Wadham J.L., and Cockell C.S. (2017) Viable cold-tolerant iron-reducing microorganisms in geographically diverse subglacial environments. Biogeosciences 14:1445–1455

[B68] *Noack L., Verseux C., Serrano P., Musilova M., Nauny P., Samuels T., Schwendner P., Simoncini E., and Stevens A. (2015) Astrobiology from early-career scientists' perspective. International Journal of Astrobiology 14:533–535

[B69] Olsson-Francis K., de la Torre R, and Cockell C.S. (2010) Isolation of novel extreme-tolerant cyanobacteria from a coastal rock-dwelling microbial community using exposure to low Earth orbit. Appl Environ Microbiol 76:2115–2121 20154120 10.1128/AEM.02547-09 PMC2849240

[B70] *O'Malley-James J.T., Greaves J.S., Raven J.S., and Cockell C.S. (2013) Swansong biospheres: refuges for life and novel microbial biospheres on terrestrial planets near the end of their habitable lifetimes. International Journal of Astrobiology 12:99–112

[B71] *O'Malley-James J.T., Cockell C.S., Greaves J.S., and Raven J.A. (2014) Swansong biospheres II: the final signs of life on terrestrial planets near the end of their habitable lifetimes. International Journal of Astrobiology 13:229–243

[B72] *O'Malley-James J.T., Greaves J.S., Raven J.A., and Cockell C.S. (2015) In search of future Earths: assessing the possibility of finding Earth analogues in the later stages of their habitable lifetimes. Astrobiology 15:400–411 25984921 10.1089/ast.2014.1229

[B73] *Payler S.J., Biddle J.F., Coates A., Cousins C.R., Cross R.E., Cullen D.C., Downs M.T., Direito S.O.L., Gray A.L., Genis J., Gunn M., Hansford G.M., Harkness P., Holt J., Josset J.-L., Li X., Lees D.S., Lim D.S.S., McHugh M., McLuckie D., Meehan E., Paling S.M., Souchon A., Yeoman L., and Cockell C.S. (2016) Planetary science and exploration in the deep subsurface: results from the MINAR Program, Boulby Mine, UK. International Journal of Astrobiology 15:333–344

[B74] *Perfumo A., Elsaesser A., Littmann S., Foster R.A., Kuypers M.M.M., Cockell C.S., and Kminek G. (2014) Epifluorescence, SEM, TEM and nanoSIMS image analysis of the cold phenotype of *Clostridium psychrophilum* at subzero temperatures. FEMS Microbiol Ecol 90:869–882 25319134 10.1111/1574-6941.12443

[B75] *Pontefract A., Osinski G.R., Cockell C.S., Moore C.A., Moores J.E., and Southam G. (2014) Impact-generated endolithic habitat within crystalline rocks of the Haughton Impact Structure, Devon Island, Canada. Astrobiology 14:522–533 24926727 10.1089/ast.2013.1100

[B76] *Pontefract A., Osinski G.R., Cockell C.S., Southam G., McLausland P.J.A., Umoh J., and Holdsworth D.W. (2016) Microbial diversity of impact-generated habitats. Astrobiology 16:775–786 27732069 10.1089/ast.2015.1393

[B77] Postberg F., Kempf S., Schmidt J., Brilliantov N., Beinsen A., Abel B., Buck U., and Srama R. (2009) Sodium salts in E-ring ice grains from an ocean below the surface of Enceladus. Nature 459:1098–1101 19553992 10.1038/nature08046

[B78] Quinlan C.L. (2015) Bringing astrobiology down to Earth. Am Biol Teach 77:567–574

[B79] *Raafat K., Burnett J., Chapman T., and Cockell C.S. (2013) The physics of mining in space. Astronomy and Geophysics 54:5.10–5.12

[B80] Race M., Denning K., Bertka C.M., Dick S.J., Harrison A.A., Impey C., and Mancinelli R. (2012) Astrobiology and society: building an interdisciplinary research community. Astrobiology 12:958–965 23046203 10.1089/ast.2011.0723 PMC3484766

[B81] Rousseau J.J. (1762) The Social Contract, France

[B82] *Samuels T., Noack L., Verseux C., and Serrano P. (2015) A new network for astrobiology in Europe. Astronomy and Geophysics 56:2.15–2.17

[B83] Sauterer R. (2000) Astrobiology courses—a useful framework for teaching interdisciplinary science. J Coll Sci Teach 29:233–234

[B84] Schulte P., Alegret L., Arenillas I., Arz J.A., Barton P.J., Bown P.R., Bralower T.J., Christeson G.L., Claeys P., Cockell C.S., Collins G.S., Deutsch A., Goldin T.J., Goto K., Grajales-Nishimura J.M., Grieve R.A.F., Gulick S.P.S., Johnson K.R., Kiessling W., Koeberl C., Kring D.A., MacLeod K.G., Matsui T., Melosh J., Montanari A., Morgan J.V., Neal C.R., Nichols D.J., Norris R.D., Pierazzo E., Ravizza G., Rebolledo-Vieyra M., Reimold W.U., Robin E., Salge T., Speijer R.P., Sweet A.R., Urrutia-Fucugauchi J., Vajda V., Whalen M.T., and Willumsen P.S. (2010) The Chicxulub asteroid impact and mass extinction at the Cretaceous-Paleogene boundary. Science 327:1214–1218 20203042 10.1126/science.1177265

[B85] *Schwieterman E.W., Cockell C.S., and Meadows V.S. (2015) Nonphotosynthetic pigments as potential biosignatures. Astrobiology 15:341–361 25941875 10.1089/ast.2014.1178 PMC4442567

[B86] *Schwieterman E.W., Kiang N.Y., Parenteau M.N., Harman C.E., DasSarma S., Fisher T.M., Arney G.N., Hartnett H., Reinhard C., Olson S.L., Meadows V.S., Cockell C.S., Walker S., Grenfell J.L., Hegde S., Rugheimer S., Hu R., and Lyons T.W. (2018) Exoplanet biosignatures: a review of remotely detectable signs of life. Astrobiology, in press, doi:10.1089/ast.2017.1729 PMC6016574 29727196

[B87] *Smith I.W.M., Cockell C.S., and Leach S. (2013) Astrochemistry and Astrobiology, Springer, Heidelberg

[B88] *Stevens A., Forgan D., and O'Malley James J. (2016) Observational signatures of self-destructive civilizations. International Journal of Astrobiology 15:333–344

[B89] *Stevenson A., Burkhardt J., and Cockell C.S. (2015) Multiplication of microbes below 0.690 water activity: implications for terrestrial and extraterrestrial life. Environ Microbiol 17:257–277 25142751 10.1111/1462-2920.12598

[B90] Tang B.L. (2005) Astrobiological themes for integrative undergraduate general science education. Astronomy Education Review 4:110–114

[B91] Taylor J.C.M. (1998) Chapter 6: Upper Permian-Zechstein. In Petroleum Geology of the North Sea: Basic Concepts and Recent Advances, 4^th^ ed., edited by Glennie K.W., Blackwell, Chichester, UK, pp 174–211

[B92] Thucydides. (BCE 431) The History of the Peloponnesian War, Athens, Greece

[B93] Vance S., Bouffard M., Choukroun M., and Sotin C. (2014) Ganymede's internal structure including thermodynamics of magnesium sulfate oceans in contact with ice. Planet Space Sci 96:62–70

[B94] *Wadsworth J. and Cockell C.S. (2017) The Janus face of iron on anoxic worlds: iron oxides are both protective and destructive to life on the Early Earth and present-day Mars. FEMS Microbiol Ecol 93, doi:10.1093/femsec/fix056 28460085

[B95] Waite J.H., Lewis W.S., Magee B.A., Lunine J.I., McKinnon W.B., Glein C.R., Mousis O., Young D.T., Brockwell T., Westlake J., Nguyen M.-J., Teolis B.D., Niemann H.B., McNutt R.L., Perry M., and Ip W.-H. (2009) Liquid water on Enceladus from observations of ammonia and ^40^Ar in the plume. Nature 460:487–490

[B96] Woodgate A., Scott A-M., Macleod H.A., and Haywood J. (2015) Differences in online study behaviour between sub-populations of MOOC learners. Educación XX1. 18.2:147–163

[B97] *Yates J.S., Palmer P.I., Biller B., and Cockell C.S. (2017) Atmospheric habitable zones in Y dwarf atmospheres. Astrophysical Journal 836, doi:10.3847/1538-4357/836/2/184

[B98] Zhang Y., Krause M., and Mutti M. (2013) The formation and structure evolution of Zechstein (Upper Permian) salt in Northeast German Basin: a review. Open Journal of Geology 3:411–426

